# Attenuation of inflammatory and neuropathic pain behaviors in mice through activation of free fatty acid receptor GPR40

**DOI:** 10.1186/s12990-015-0003-8

**Published:** 2015-02-12

**Authors:** Prasanna Karki, Takashi Kurihara, Tomoya Nakamachi, Jun Watanabe, Toshihide Asada, Tatsuki Oyoshi, Seiji Shioda, Megumu Yoshimura, Kazunori Arita, Atsuro Miyata

**Affiliations:** Department of Neurosurgery, Graduate School of Medical and Dental Sciences, Kagoshima University, 8-35-1 Sakuragaoka, Kagoshima City, Kagoshima 890-8544 Japan; Department of Pharmacology, Graduate School of Medical and Dental Sciences, Kagoshima University, 8-35-1 Sakuragaoka, Kagoshima City, Kagoshima 890-8544 Japan; Department of Anatomy, Showa University, School of Medicine, 1-5-8 Hatanodai, Shinagawa-ku Tokyo, 142-8555 Japan; Laboratory of Regulatory Biology, Graduate School of Science and Engineering, University of Toyama, 3190-Gofuku, Toyama, 930-8555 Japan; Graduate School of Health Sciences, Kumamoto Health Science University, 325 Izumi-machi, Kumamoto, 861-5598 Japan

**Keywords:** Allodynia, Carrageenan, Complete Freund’s adjuvant, FFA1, FFAR1, Hyperalgesia, Spinal cord, Spinal nerve ligation, Whole-cell patch-clamp

## Abstract

**Background:**

The G-protein-coupled receptor 40 (GPR40) is suggested to function as a transmembrane receptor for medium- to long-chain free fatty acids and is implicated to play a role in free fatty acids-mediated enhancement of glucose-stimulated insulin secretion from pancreas. However, the functional role of GPR40 in nervous system including somatosensory pain signaling has not been fully examined yet.

**Results:**

Intrathecal injection of GPR40 agonist (MEDICA16 or GW9508) dose-dependently reduced ipsilateral mechanical allodynia in CFA and SNL models and thermal hyperalgesia in carrageenan model. These anti-allodynic and anti-hyperalgesic effects were almost completely reversed by a GPR40 antagonist, GW1100. Immunohistochemical analysis revealed that GPR40 is expressed in spinal dorsal horn and dorsal root ganglion neurons, and immunoblot analysis showed that carrageenan or CFA inflammation or spinal nerve injury resulted in increased expression of GPR40 in these areas. Patch-clamp recordings from spinal cord slices exhibited that bath-application of either MEDICA16 or GW9508 significantly decreased the frequency of spontaneous excitatory postsynaptic currents in the substantia gelatinosa neurons of the three pain models.

**Conclusions:**

Our results indicate that GPR40 signaling pathway plays an important suppressive role in spinal nociceptive processing after inflammation or nerve injury, and that GPR40 agonists might serve as a new class of analgesics for treating inflammatory and neuropathic pain.

**Electronic supplementary material:**

The online version of this article (doi:10.1186/s12990-015-0003-8) contains supplementary material, which is available to authorized users.

## Background

The G-protein-coupled receptor 40 (GRP40) is a seven-transmembrane domain receptor, which is demonstrated to be predominantly expressed in pancreatic β-cells and activated by medium- to long-chain (C12-C22) free fatty acids (FFAs) [1-5]. Because FFAs have been long recognized as important regulators of glucose homeostasis via their ability to stimulate insulin secretion in the presence of glucose, GPR40 became a promising therapeutic target for type 2 diabetes treatment since its deorphanization, and a number of small-molecule GPR40 agonists are under development as drugs for type 2 diabetes [4,6]. In contrast, progress toward understandings of the physiological role of GPR40 in other fields has been relatively slower.

Based on mRNA measurements and immunohistochemical analyses, GPR40 expression has been documented in several tissues including human, primate, and rodent central nervous system (CNS) [1,7-9]. However, its role in the CNS has been largely unclarified. Since the spinal cord has been shown to be one of the areas which express abundant GPR40 mRNA and protein among the CNS regions, we have tested a possibility in the present study that GPR40 plays a role in the regulation of spinal nociceptive signaling. We first examined the distribution of GPR40 protein in mouse spinal cord and dorsal root ganglia and then quantified the expression of this protein after peripheral inflammation or nerve injury as reported in our preliminary reports [10,11]. Next, we have tested the effects of GPR40 agonists and an antagonist on the mouse inflammatory and neuropathic pain-like behaviors. Finally, we have analyzed the effects of the GPR40 agonists on the excitatory synaptic transmission in the superficial dorsal horn neurons in acute adult mouse spinal cord slices to evaluate cellular mechanisms.

## Results

### Expression of GPR40 in mouse spinal cord and dorsal root ganglia (DRGs)

Although GPR40 expression in the human and primate spinal cords is demonstrated to be even higher than in the pancreas [1,7], there is still no clear immunohistochemical demonstration of GPR40 expression in the mouse spinal cord. Furthermore, to the best of our knowledge, there have been no reports regarding the expression of GPR40 in the mouse primary sensory neurons. Thus, we first attempted to verify the expression of GPR40 by immunoblot, and then to characterize the distribution of GPR40-positive cells in the mouse spinal cord and DRGs by immunohistochemical technique.

**Figure 1:**
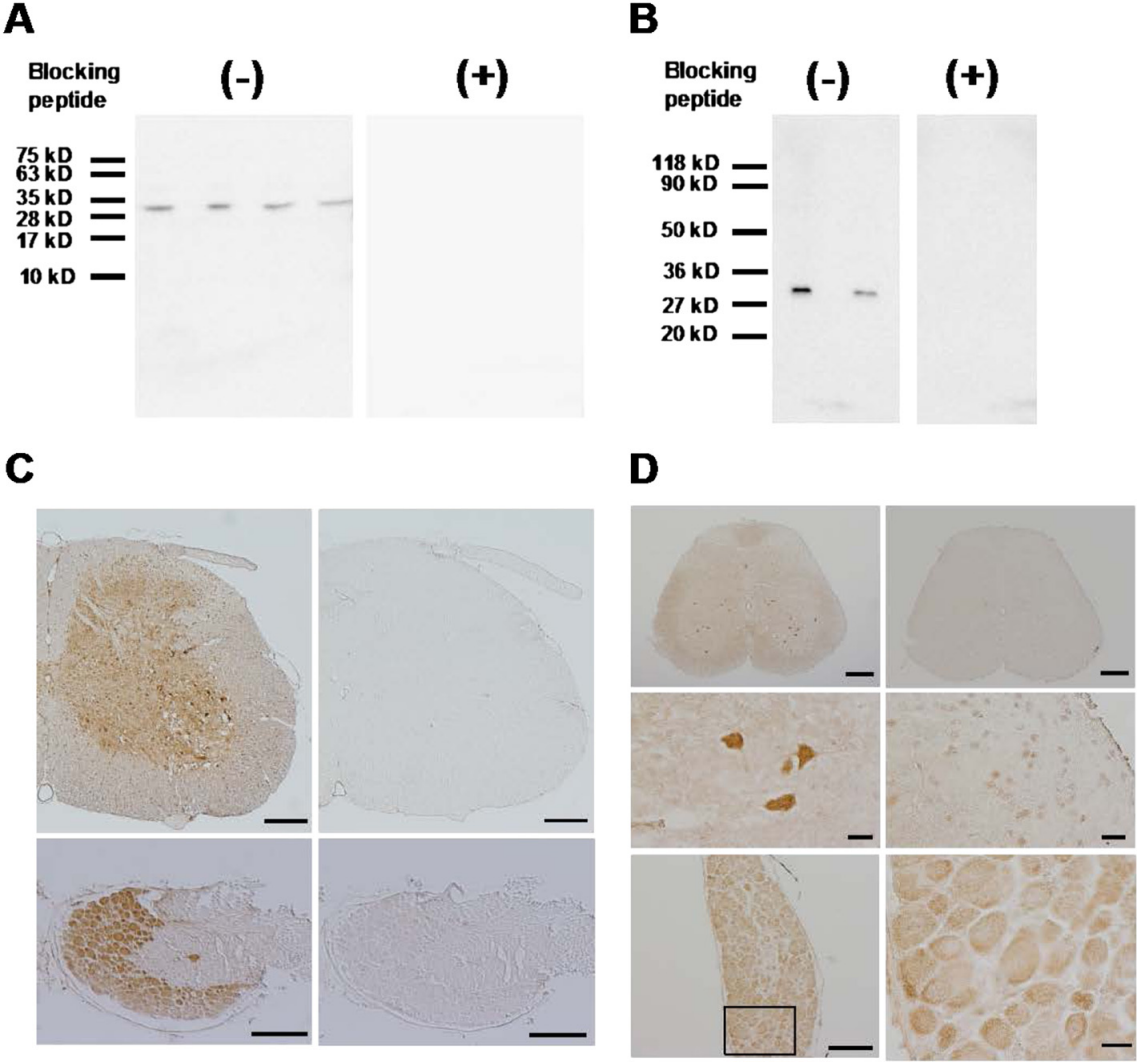
**Expression of GPR40 in spinal cord and dorsal root ganglia (DRGs). (A, B)** Representative western blots (left panels) performed with two different goat polyclonal anti-GPR40 antibodies (**A**: C-17; **B** Y-17). These western blot bands were abolished by the antibody preabsorption with respective antigen peptides (right panels). Each band in both blot was from whole lumbar spinal cord (L3-5) sample from a different animal. **(C, D)** Representative macrographs of naïve lumbar spinal cords and L4 DRGs labelled with C-17 **(C)** or Y-17 **(D)** antibody. These immunostaining patterns (**C**, left panels; **D**, top left) were disappeared by preabsorption (**C**, right panels; **D**, top right). Ventral horn of naïve spinal cord showed relatively intense immunoreactivities in several cells (**D**, middle left). The dorsal horn of naive spinal cord showed that GPR40-positive cells were broadly distributed (**D**, middle right). In naïve L4 DRG, GPR40 appeared to be expressed in neurons with no specificity regarding cell size (**D**, bottom left). Boxed area was enlarged in the bottom right panel. Scale bar = 200 μm (**C** and **D** top panels); 20 μm (**D**, middle panels and bottom right panel); 100 μm (**D**, bottom left panel).

In this study, we used two commercially available GPR40 antibodies (C-17 and Y-17). We first confirmed that both antibodies exhibited same single band with expected molecular weight (31 kDa) in immunoblot analyses and the specificity of each staining was corroborated by antigen absorption test with each blocking peptide (Figure 1A,B).

**Figure 2:**
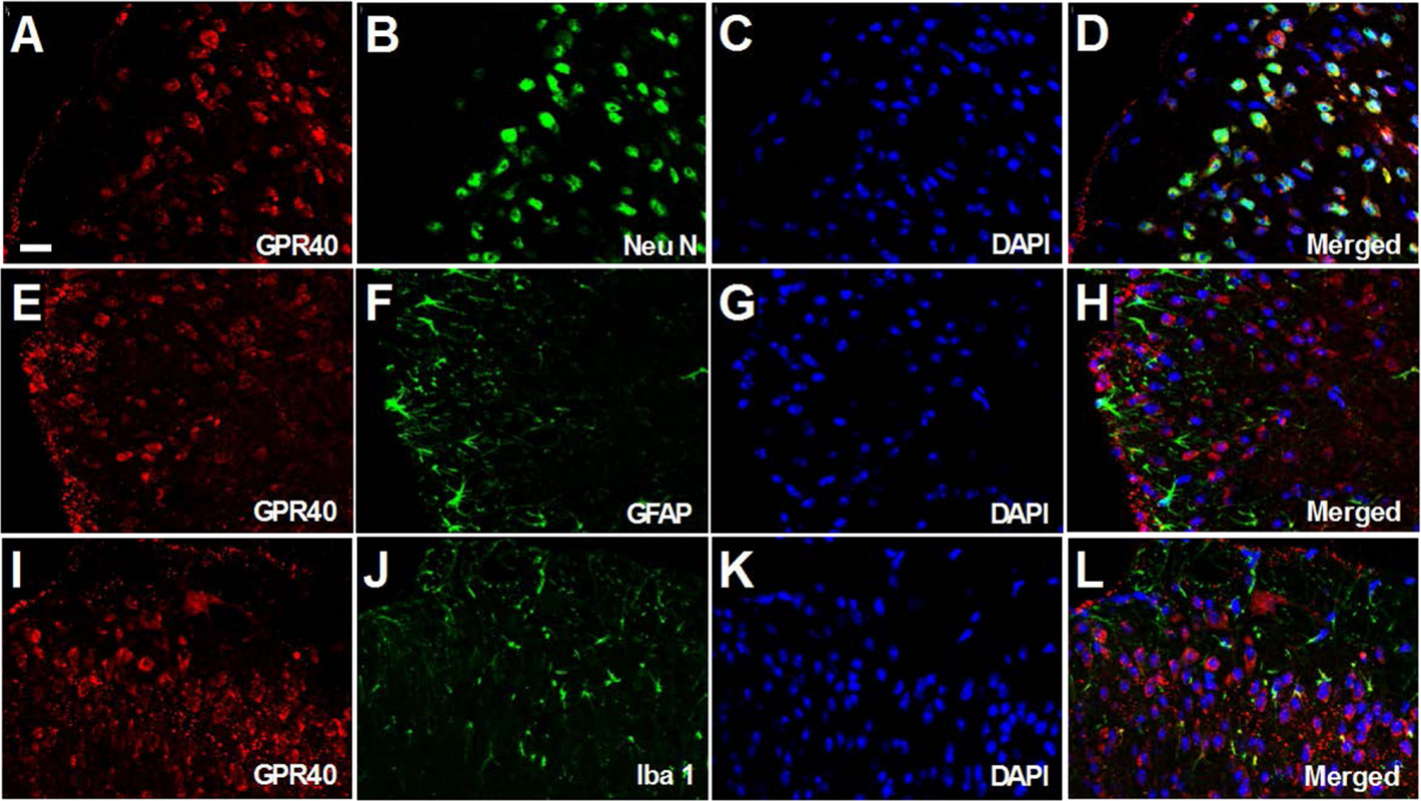
**Double immunofluorescence staining performed with Y-17 anti-GPR40 antibody using naïve lumbar spinal dorsal horn.** Double-staining for GPR40 (red) and NeuN, GFAP or Iba 1 (green) with DAPI (blue) showed GPR40 immunoreactivity mostly colocalized with a neuronal marker, NeuN **(B)**, but rarely with an astrocytic marker, GFAP **(F)** or a microglial marker, Iba 1 **(J)** in the spinal dorsal horn **(A-L)**. Scale bar = 20 μm.

In naïve spinal cord, GPR40-positive cells were shown to be widely distributed not only in the dorsal horn, but also in the ventral horn (Figure 1C,D). Double staining experiments revealed that GPR40 immunoreactivity (GPR40-IR) was predominantly colocalized with a neuronal marker, neuronal specific nuclear protein (NeuN) (Figure 2A-D), but rarely with an astrocytic marker, glial fibrillary acid protein (GFAP) (Figure 2E-H), and a microglial marker, ionized calcium binding adaptor molecule 1 (Iba 1) (Figure 2I-L). In naïve DRGs, GPR40-IR was also appeared to be mainly observed in neurons with no apparent preference with regard to cell size (Figure 1C,D). In both spinal cord and DRGs, the two antibodies showed similar staining pattern, which was abolished by the antibody preabsorption with respective immunogenic peptide (Figure 1C,D).

### Peripheral inflammation or nerve injury upregulates GPR40 expression both in the spinal cord and DRGs

**Figure 3:**
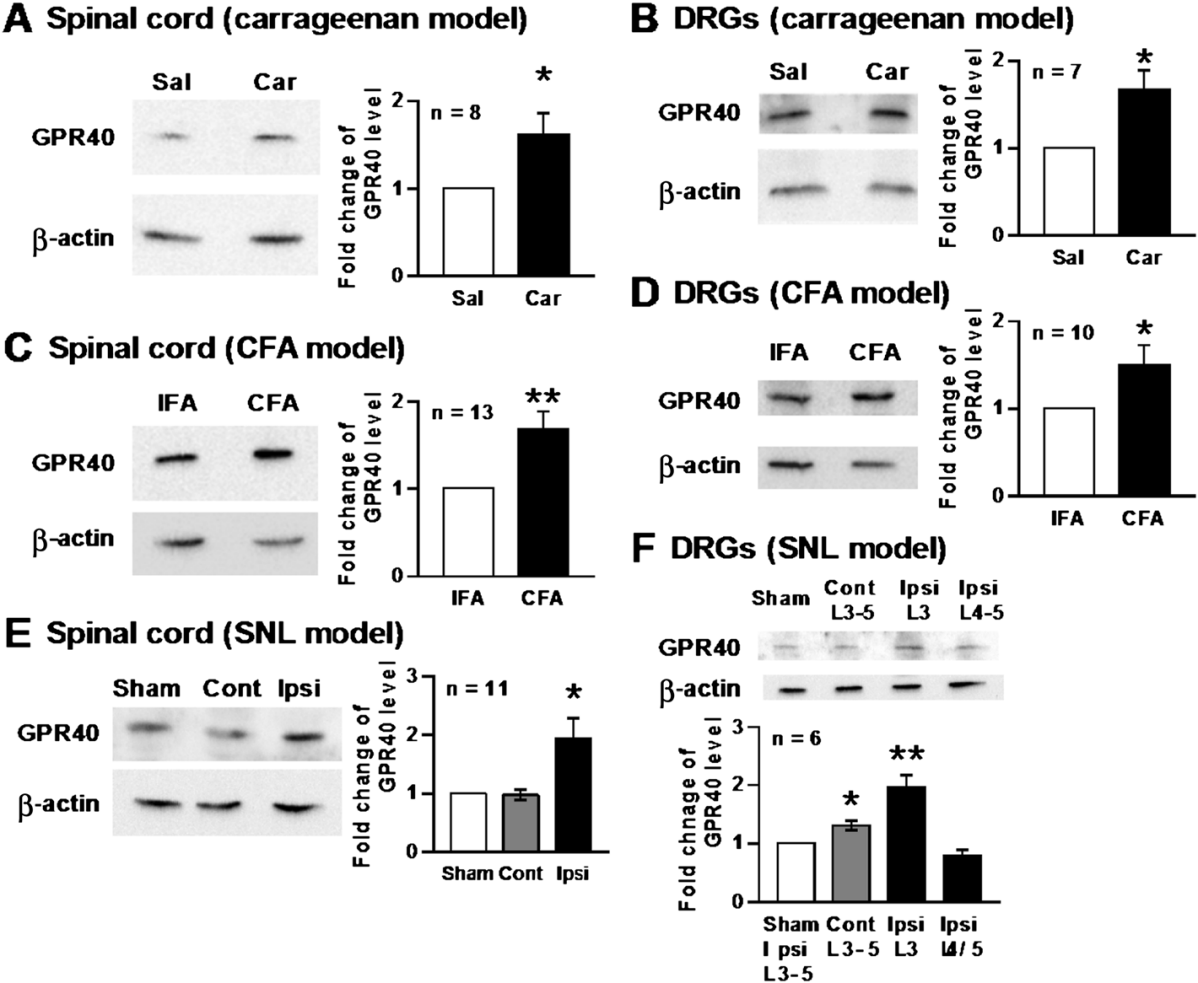
**Carrageenan-, CFA- or SNL-treatment upregulates expression level of GPR40.** Representative western blots showing that the levels of GPR40 protein expression in the spinal cord **(A, C, E)** and dorsal root ganglia (DRGs; **B, D, F**) were upregulated by carrageenan **(A, B)**, CFA **(C, D)** or SNL **(E, F)** treatment. L3-5 spinal segments and DRGs ipsilateral to carrageenan (Car) or CFA, or both ipsi- and contralateral to SNL treatment were dissected 6 hours after Car, 3 days after CFA and 2–3 weeks after SNL treatment, respectively. As a control, saline (Sal) and incomplete Freund’s adjuvant (IFA) were injected instead of Car and CFA, respectively, and for SNL, samples after sham operation (Sham) were employed. The GPR40/β-actin ratio of each control was set at 1 for quantification. Fold change of GPR40, expressed as mean ± SEM, was shown in the graph to the right or beneath each gel image. ^*^ *P* < 0.05 and ^**^ *P* < 0.01 (Student’s *t*-test).

We next assessed the expression levels of GPR40 in the spinal cord and DRGs in peripheral inflammation and nerve injury. Immunoblot studies showed that expression levels of GPR40 were significantly upregulated in both spinal cord and DRGs after carrageenan (6 hours)- (Figure 3A,B) or CFA (3 days)- (Figure 3C,D) treatment. In SNL (2–3 weeks) injury model, the expression level of GPR40 was also significantly enhanced in spinal cord ipsilateral to the injury (Figure 3E). Interestingly, the expression of GPR40 was not changed in the injured L4/5 DRGs, but significantly increased in the adjacent L3 DRG and contralateral (L3-5) DRGs, although the change in the contralateral one was considerably less extensive (Figure 3F).

**Figure 4:**
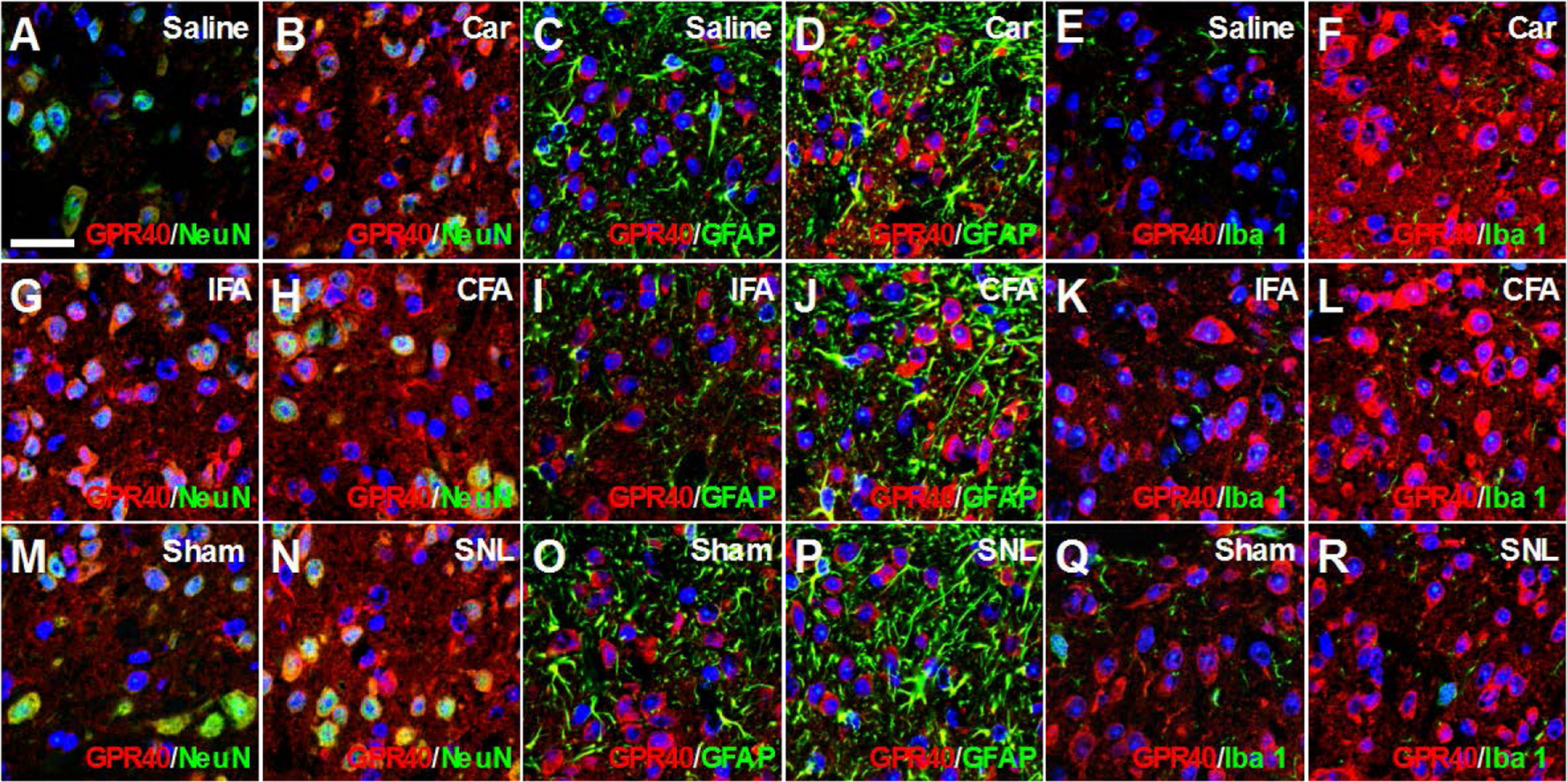
**Effects of peripheral inflammation or spinal nerve injury on the GPR40 expression in the superficial spinal dorsal horn.** Double-staining for GPR40 (red) and NeuN, GFAP or Iba 1 (green) with DAPI (blue) indicated that GPR40 immunoreactivity still mainly colocalized with a neuronal marker, NeuN **(A, B, G, H, M, N)**, but rarely with an astrocytic marker, GFAP **(C, D, I, J, O, P)** or a microglial marker, Iba 1 **(E, F, K, L, Q, R)** in the spinal dorsal horn even after the carrageenan (Car) **(A-F)**, complete Freund’s adjuvant (CFA) **(G-L)** or spinal nerve ligation (SNL) **(M-R)** treatment. Lumbar spinal cords (L4-5) were dissected 6 hours after carrageenan, 3 days after CFA, and 2–3 weeks after SNL treatment. As each control, saline, incomplete Freund’s adjuvant (IFA) and sham-treatment were employed, respectively. C-17 anti-GPR40 antibody was used. Scale bar = 25 μm.

**Figure 5:**
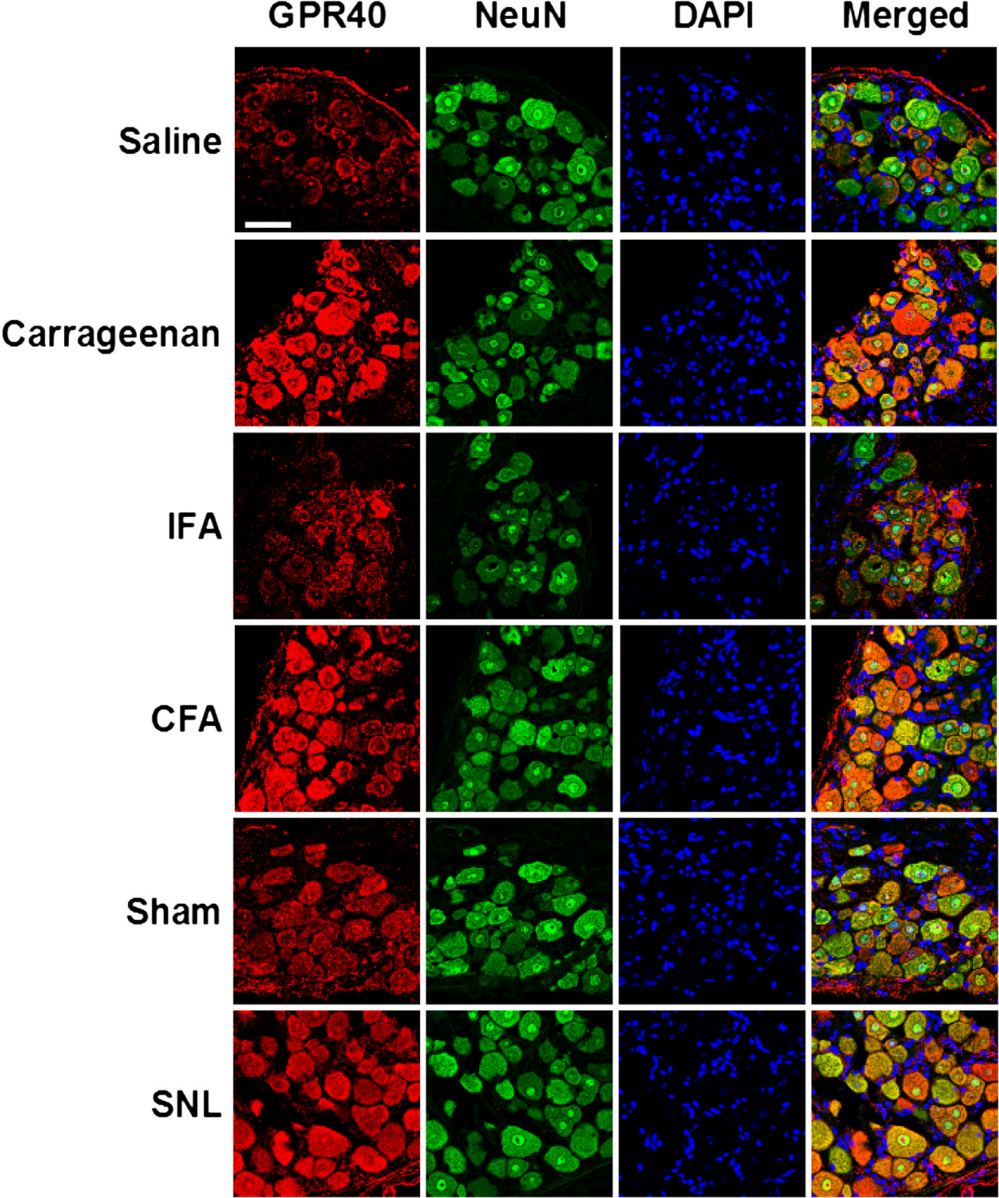
**Effects of peripheral inflammation or spinal nerve injury on the GPR40 expression in the dorsal root ganglion (DRG) neurons.** Double-staining for GPR40 (red) and NeuN (green) with DAPI (blue) indicated that GPR40 immunoreactivity mainly colocalized with a neuronal marker, NeuN in the DRGs even after the carrageenan, complete Freund’s adjuvant (CFA) or spinal nerve ligation (SNL) treatment. Lumbar DRGs (L4) were dissected 6 hours after carrageenan, 3 days after CFA, and 2–3 weeks after SNL treatment. As each control, saline, incomplete Freund’s adjuvant (IFA) and sham-treatment were employed, respectively. C-17 anti-GPR40 antibody was used. Scale bar = 50 μm.

Immunohistochemical analyses suggested that the distribution patterns of GPR40-IR in both spinal cords and DRGs were not largely affected, but the expression levels appeared to be increased after carrageenan, CFA or spinal nerve injury (Figures 4 and 5).

These results suggested that the expression of GPR40 mainly increased in neurons but not in astrocytes or microglial cells after inflammation or nerve injury.

### Intrathecal injection of GPR40 agonist (MEDICA16 or GW9508) attenuated inflammatory and neuropathic pain-like behaviors

**Figure 6:**
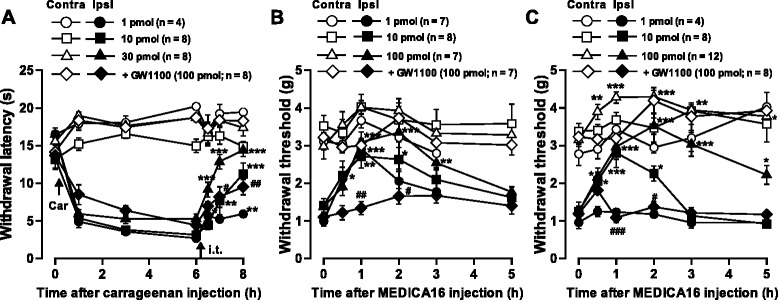
**Effects of MEDICA16 on inflammatory and neuropathic pain-like behaviors. (A)** Effect of intrathecal (i.t.) injection of MEDICA16 on carrageenan (Car)-induced thermal hyperalgesia. The GPR40 agonist, MEDICA 16, was injected 6 hours after carrageenan injection. **(B, C)** Effects of intrathecal injection of MEDICA16 on CFA **(B)**- and SNL **(C)**-induced mechanical allodynia. Paw withdrawal latency to thermal stimuli **(A)** or paw withdrawal threshold to mechanical stimulation **(B, C)** are plotted against the time after carrageenan injection into a hindpaw **(A)** or after intrathecal injection of MEDICA16 with or without GW1100. Data are mean ± SEM. ^*^ *P* < 0.05, ^**^ *P* < 0.01, ^***^ *P* < 0.001, compared with pre-drug (at 6 hours in **A** and at 0 hour in **B** and **C**) data (one-way ANOVA followed by Dunnett’s post hoc test). ^#^ *P* < 0.05, ^##^ *P* < 0.01, ^###^ *P* < 0.001, compared with the 30 pmol group in **(A)** and the 10 pmol group in **(B)** and **(C)** (Student's *t*-test).

Since GPR40 was extensively localized in both spinal dorsal horn and DRGs in a normal state and upregulated after peripheral inflammation or nerve injury, we then evaluated the role of GPR40 in spinal nociception using both naïve control and the mouse models of inflammatory (carrageenan and CFA) and neuropathic (SNL) pain. In naïve mice, intrathecal (i.t.) administration of a high dose of each GPR40 agonist, MEDICA16 (100 pmol; [3,12,13]) and GW9508 (30 pmol; [12,14]) did not show significant effects on mechanical and thermal sensitivities of hindpaws within 2 hours observation time (Additional file 1). In marked contrast, i.t. application of MEDICA16 dose-dependently (1–30 pmol) increased withdrawal latency of the hindpaw ipsilateral to carrageenan (Figure 6A). I.t. injection of GW9508 (30 pmol), also effectively attenuated the thermal hyperalgesia induced by carrageenan (Additional file 2A). These anti-hyperalgesic effects of GPR40 agonists were significantly reversed by a GPR40 antagonist, GW1100 (100 pmol; [14,15]) (Figure 6A, Additional file 2B).

Similarly, i.t. administration of MEDICA16 dose-dependently increased withdrawal threshold of the hindpaw ipsilateral to CFA-induced inflammation (Figure 6B) and SNL-induced nerve injury (Figure 6C), and these anti-allodynic effects of MEDICA16 were significantly antagonized by GW1100 (100 pmol; Figure 6B,C). Intriguingly, 100 pmol of MEDICA16 significantly increased contralateral threshold in SNL injury model (Figure 6C).

Unlike these anti-allodynic and anti-hyperalgesic effects, motor function in the rotarod test was not affected by i.t. administration of MEDICA16 (100 pmol) or GW9508 (30 pmol) (Additional file 3A). Lastly, possible hypoglycemic action by GPR40 agonists was evaluated by measuring blood glucose levels. As shown in Additional file 3B, glucose levels were not changed after i.t. administration of each GPR40 agonist.

### I.t. injection of a GPR40 antagonist, GW1100, decreased the withdrawal thresholds of contralateral hindpaws in CFA and SNL pain models

**Figure 7:**
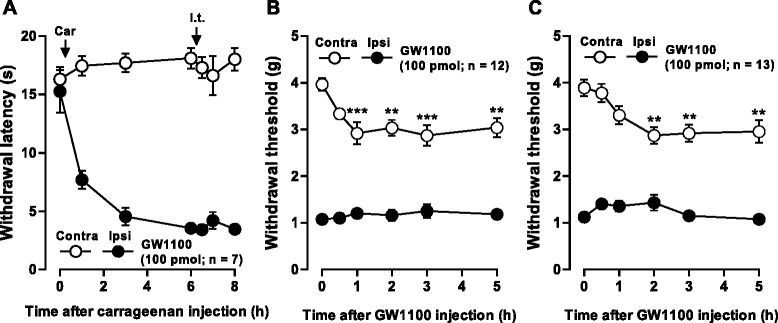
**Effects of GW1100 on inflammatory and neuropathic pain-like behaviors. (A)** Effect of intrathecal (i.t.) injection of GW1100 on carrageenan (Car)-induced thermal hyperalgesia. The GPR40 antagonist, GW1100, was injected 6 hours after carrageenan injection. **(B, C)** Effects of intrathecal injection of GW1100 on CFA **(B)**- and SNL **(C)**-induced mechanical allodynia. Paw withdrawal latency to thermal stimuli **(A)** or paw withdrawal threshold to mechanical stimulation **(B, C)** are plotted against the time after carrageenan injection into a hindpaw **(A)** or after intrathecal injection of GW1100 **(B, C)**. Data are mean ± SEM. ^**^ *P* < 0.01, ^***^ *P* < 0.001, compared with pre-drug (at 0 hour) data (one-way ANOVA followed by Dunnett’s post hoc test).

To investigate whether endogenous GPR40 ligands are involved in the regulation of inflammatory and neuropathic pain states, we evaluated the effects of GW1100 in the three pain models. Although i.t. injection of GW1100 (100 pmol) had no significant effects on the withdrawal latencies of the both hindpaws in the carrageenan-induced inflammatory pain model (Figure 7A), the same dosage of GW1100 significantly decreased the withdrawal threshold of the hindpaw contralateral to CFA-induced inflammation (Figure 7B) and SNL-induced nerve injury (Figure 7C) without affecting their ipsilateral hindpaw thresholds.

### GPR40 agonists decreased the frequency of sEPSCs in the spinal dorsal horn neurons of inflammatory and neuropathic pain model mice

To explore the mechanism of the antinociception induced by i.t. administration of GPR40 agonists, we prepared lumbar (mainly L4) spinal cord slices from adult mice (7–9 weeks old), and performed patch-clamp recordings from lamina II substantia gelatinosa (SG) neurons ipsilateral to carrageenan, CFA, SNL or control (vehicle injection or sham operation) treatment [16-18]. The SG neurons of the spinal dorsal horn play an important role in the transmission and modulation of nociceptive information from the periphery to the CNS [19,20], and are one of the key sites generating synaptic plasticity (central sensitization) after tissue injury [21-23]. Such plasticity is exhibited in part as changes in spontaneous excitatory postsynaptic currents (sEPSCs), which could point out both presynaptic mechanisms (frequency changes) and postsynaptic mechanisms (amplitude changes) [16,24-28].

We first examined the passive membrane properties of SG neurons, all of which had resting potentials more negative than −60 mV in control, inflammatory and neuropathic pain model mice. No differences were found in the resting membrane potential and input membrane resistance among the groups (Additional file 4).

Next we characterized sEPSCs, which were recorded under voltage-clamp at a holding potential of −70 mV, from control and pain model mice (Additional file 5). The mean amplitude of sEPSCs was not significantly different among the groups. The mean frequency of sEPSCs, on the other hand, was significantly different. We observed that the average frequency of sEPSCs was significantly increased in mice inflamed with CFA 3 days before, although carrageenan inflammation and SNL injury did not change average sEPSC frequency.

**Figure 8:**
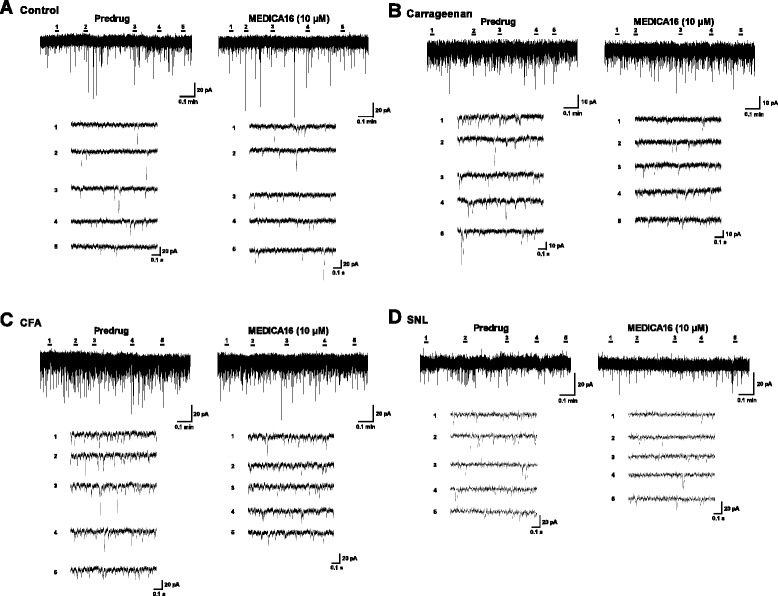
**MEDICA16 decreased the mean frequency of sEPSCs in inflammatory and neuropathic pain model mice.** Representative traces of sEPSCs in SG neurons of the spinal cord slices from control **(A)**, carrageenan **(B)**-, CFA **(C)**- and SNL **(D)**-treated mice showing the effects of MEDICA16 (10 μM). Lower five traces represent sEPSCs at five given points in time presented above the upper trace, and are shown in an expanded time scale.

**Figure 9:**
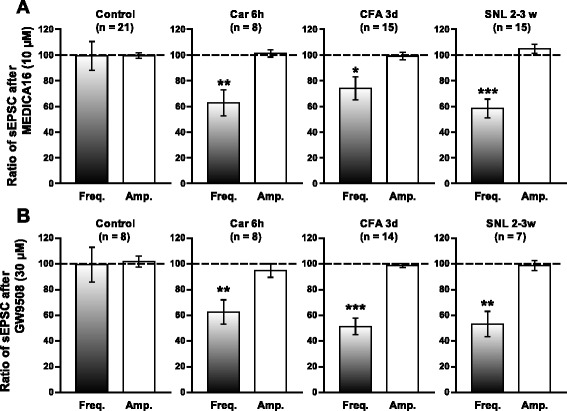
**Summary of results, testing the effects of MEDICA16 (A) and GW9508 (B) on the sEPSC frequencies and amplitudes.** The percentage compared to pre-drug response (as 100%) was shown as % control. ^*^ *P* < 0.05, ^**^ *P* < 0.01, compared with pretreatment control (Student’s *t* test).

We then examined the possibility that the observed effects of the two GPR40 agonists originate from the regulation of the excitatory synaptic transmission in lamina II SG neurons. In superfusion of spinal cord slices from control mice, MEDICA16 (10 μM) and GW9508 (30 μM) altered neither the mean frequency nor the mean amplitude of sEPSCs (Figure 8A, Additional file 6A). In contrast, both GPR40 agonists significantly suppressed the average sEPSC frequencies without affecting the average sEPSC amplitudes recorded from carrageenan (Figure 8B, Additional file 6B)-, CFA (Figure 8C, and Additional file 6C)-, and SNL (Figure 8D, Additional file 6D)-treated mice (Figure 9). It should be noted, however, that the changes in sEPSC frequency induced by GPR40 agonists did not seem to be homogenous across the tested population of neurons in control group (Table 1, Additional file 7). In this group, 6 out of the 21 neurons and 3 out of the 8 neurons showed an increase of the sEPSC frequency, 12 of the 21 neurons and 3 of the 8 neurons showed a decrease, whereas in 3 and 2 neurons, the frequency did not change after MEDICA16 (10 μM) and GW9508 (30 μM) application, respectively. Thus, although GPR40 is considered to be functional in control mice, the excitatory and inhibitory effects of GPR40 agonists may cancel each other in SG neuronal circuitry, leading to apparent non-significant effects of i.t. GPR40 agonists in pain behavioral assay. Intriguingly, peripheral inflammation and nerve injury turned these agonists mainly effective in decreasing mean frequencies of sEPSCs in SG neurons. Furthermore, in SNL mice, we have encountered several other SG neurons, in which clear outward currents were induced by MEDICA16 (10 μM: Additional file 8). Incidentally, we have never observed MEDICA16 (10 μM)-induced outward currents in control, carrageenan (6 h) and CFA (3d) SG neurons so far (data not shown).

**Table 1:** **Effects of GPR40 agonists on sEPSC frequency**

	**MEDICA16 (10 μM)**			**GW9508 (30 μM)**		
	**Increase**	**Decrease**	**No change**	**Increase**	**Decrease**	**No change**
**Control**	156.6 ± 25.5 (6/21)	69.3 ± 4.5^***^(12/21)	104.9 ± 1.2 (3/21)	136.0 ± 15.1 (3/8)	61.7 ± 9.2 (3/8)	101.8 ± 6.5 (2/8)
**Carrageenan 6 h**	― (0/8)	52.6 ± 10.6^**^ (6/8)	92.9 ± 1.6 (2/8)	― (0/8)	56.0 ± 7.7^**^ (7/8)	109.9 (1/8)
**CFA 3d**	133.3 ± 12.0 (3/15)	59.1 ± 4.6^***^(12/15)	― (0/15)	― (0/14)	51.4 ± 6.3^***^(14/14)	― (0/14)
**SNL 2–3 w**	113.8 (1/15)	54.4 ± 6.5^***^(14/15)	― (0/15)	― (0/7)	53.3 ± 9.8^**^ (7/7)	― (0/7)

CFA, complete Freund’s adjuvant, SNL, spinal nerve ligation.

Increase or decrease means the increase or decrease of 10% or more in the sEPSC frequency, respectively.

Values are expressed as mean ± SEM (%) and the proportion of neurons exhibiting increase, decrease or no change in parentheses.

^**^ *P* < 0.01, ^***^ *P* < 0.001, when compared with pretreatment control (Student’s *t* test).

## Discussion

In the present study, we have shown several lines of evidence that GPR40 plays a crucial regulator of spinal nociceptive signaling and sensitization in models of inflammatory and neuropathic pain. First, GPR40 was expressed in both naïve primary sensory and spinal dorsal horn neurons and its protein expression in these areas was upregulated by peripheral inflammation or nerve injury. Second, i.t. administration of GPR40 agonists effectively ameliorated behavioral hypersensitivities induced by the peripheral inflammation or nerve injury. From *in vitro* whole-cell patch-clamp studies, a part of the anti-nociceptive mechanisms was suggested to be due to the inhibitory effects of the GPR40 agonists on excitatory synaptic transmission within SG neurons of the inflamed or nerve-injured mice. These results suggest that activation of GPR40 signaling at the spinal level appeared to be effective in reduction of peripheral inflammation or nerve injury-induced pain.

### Site of action

To our knowledge, this is the first report suggesting that spinal activation of GPR40 signaling pathway is promising method to alleviate both inflammatory and neuropathic pain symptoms, although we could not eliminate a possibility that the GPR40 agonists applied intrathecally also act supraspinally to produce analgesic effects. Recent reports demonstrated that intracerebroventricular injection of GW9508 and a putative endogenous GPR40 ligand, docosahexaenoic acid, significantly reduced formalin-induced nociceptive behavior [8] and CFA-induced mechanical allodynia and thermal hyperalgesia at day 7 [29].

### Upregulation of GPR40 after unilateral peripheral inflammation or nerve injury

The mechanism of GPR40 upregulation is presently unknown, and further study is necessary. However, it may be worth noting here that there is increasing evidence suggesting that DRG neurons with intact axons also show an alteration of excitability and gene expression after peripheral nerve injury, and these changes might have functional roles in evoked neuropathic pain [30-33], because the transmission of peripheral input relies largely on DRG neurons with uninjured peripheral axons. One plausible hypothesis for such alterations is due to the neuroinflammatory responses induced by Wallerian degeneration after peripheral nerve injury [34-37]. When L4 and L5 spinal nerves are tightly ligated and injured, the axons distal to the injury undergo Wallerian degeneration. In the peripheral nerve, axons from intact L3 DRG are close proximity to degenerating axons and thus are exposed to diffusible factors released into the endoneurial space or at the nerve terminals. Thus, it would be quite possible that the inflammatory milieu may contribute to the GPR40 upregulation in L3 DRG. The upregulation of GPR40 in carrageenan and CFA inflammation may also attributable to increased production of some signaling molecules, such as cytokines and growth factors, in association with peripheral inflammation.

There is also growing evidence that unilateral nerve damage results in bilateral changes in neurochemical and electrophysiological parameters in DRGs, although it has been generally accepted that contralateral responses are usually quantitatively smaller in magnitude [38-41]. These changes are also suggested to be accompanied by neuroinflammatory responses of Wallerian degeneration [42].

We could postulate that the contralateral upregulation of GPR40 in DRGs of SNL mice would be compatible with the following two observation: (1) the highest dosage of MEDICA16 (100 pmol), employed in this study, significantly increased contralateral mechanical threshold; (2) the GPR40 antagonist, GW1100, on the contrary, caused significant contralateral mechanical hypersensitivity (see below). Similar tendency was also observed in CFA mice, although we did not examine the expression level of GPR40 in DRGs contralateral to CFA injection in this study. The pathophysiological implication of the different responses among the three pain models after the application of GPR40 agonist or antagonist are currently unclear, but it would be interesting to hypothesize that prolonged inflammation or nerve injury could activate broad compensatory mechanism to counteract pain exacerbation.

### Possible endogenous ligands

Intriguingly, i.t. application of the GPR40 antagonist, GW1100, induced mechanical hypersensitivities contralateral to CFA or SNL treatment, suggesting endogenous ligands for GPR40, which might be produced in response to persistent inflammation or nerve injury, contribute to maintain the mechanical thresholds of contralateral hindpaws, although the amount of endogenous ligands might be insufficient enough to increase ipsilateral thresholds. At present, the identities of the endogenous ligands for GPR40 responsible for the regulation of spinal nociceptive transmission in chronic pain states remain to be determined. Further rigorous study would be required to identify such ligands, since GPR40 is shown to bind a broad range of structurally and chemically distinct ligands including medium to long chain (C12-C22) FFAs and particular fatty acid metabolites [4].

### Inhibition of pain-related synaptic plasticity by GPR40 agonists

Because i.t. injection of the GPR40 agonists (MEDICA16 and GW9508) reversed both mechanical and thermal nociceptive behaviors after peripheral inflammation and nerve injury, we investigated whether bath-application of these agonists have any effects on sEPSCs by using the whole-cell patch-clamp method in SG neurons of adult spinal cord slices.

First, we characterized the effects of peripheral inflammation or nerve injury on the sEPSCs. In accordance with our previous report [16], CFA, but not carrageenan, inflammation elicited significant increase in mean frequency, but not amplitude, of sEPSCs (see ref. [16] for further discussion). In SNL model, we did not observe significant changes in frequency and amplitude of sEPSCs, which are also consistent with a previous report showing no alteration in frequency and amplitude of sEPSCs 2 weeks after partial or complete nerve injury [43].

In this study, we found that bath-application of MEDICA16 or GW9508 had no significant effect on the average sEPSC frequency and amplitude from control mice, but peripheral inflammation and nerve injury turned the sEPSC frequency to be largely decreased after the application of either GPR40 agonist. These observation suggest that the nature of GPR40 signaling in the SG neurons of inflamed or nerve-injured mice seems to be very different from those found in control animals. Similar specific effects of compounds on excitatory responses in spinal dorsal horn in pain model animals are reported in our previous studies [16,44] and also by other groups [26,45,46].

### Possible mode of action and signaling mechanisms downstream of GPR40

The preferential effects of MEDICA16 and GW9508 on the frequency of sEPSCs might suggest pre- rather than post-synaptic site of action of these drugs in SG synapses and this inhibitory modulation would contribute, at least partly, to the antinociceptive effects on inflamed or nerve-injured mice. It is generally believed that changes in the frequency and amplitude of sEPSCs are mediated by respective pre- and post-synaptic mechanisms [16,24-28]. We previously suggested that similar mechanism would be involved in the antinociceptive effects of casein kinase 1 inhibitors on neuropathic pain as well as inflammatory pain-like behaviors [16,44].

Accumulating lines of evidence have now suggested that G-protein-coupled receptors can couple to multiple heterotrimeric G proteins as well as to G-protein-independent, β-arrestin-dependent pathways to promote the activation of numerous signaling pathways in a ligand- and context-dependent manner. This new notion, referred to as biased agonism [47,48], might explain at least a part of potential mechanisms underlying present GPR40-mediated analgesic effects. As mentioned above, GPR40 binds a broad range of structurally and chemically distinct ligands, and several reports suggests that GPR40 can couple to Gα_i_ [2,49] and Gα_s_ [50] as well as G_q/11_, with which GPR40 is known to mainly couple in pancreatic β-cells to regulate insulin secretion [4]. Although we could not presently eliminate possible involvement of indirect action of GPR40 agonists in current behavioral and electrophysiological studies, it would be interesting to hypothesize that particular endogenous ligands for GPR40, which were produced in response to prolonged peripheral inflammation or nerve injury, might stabilize a specific GPR40 conformation that activate Gα_i_ pathways leading to direct inhibitory modulation of spinal synaptic transmission.

## Conclusions

In summary, our present study suggests that peripheral inflammatory and nerve injuries induced quantitative and functional alterations of GPR40 in sensory and spinal dorsal horn neurons, and that activation of GPR40 may constitute an endogenous mechanism that inhibit inflammatory and neuropathic pain states. Thus, GPR40 could be a promising therapeutic target for new analgesic drug development.

## Methods

### Animals

Male C57BL/6 J mice (5 weeks old) were purchased from Clea Japan, Inc. (Tokyo, Japan) and housed under controlled temperature (24 ± 1°C) and humidity (55 ± 10%) with a 12-hour light–dark cycle with food and water freely available. The animal experiments were approved by the Animal Care Committee of Kagoshima University (approval No. MD13074), and were conducted in accordance with the ethical guidelines for the study of experimental pain in conscious animals of the International Association of the Study of Pain.

### Animal models and behavioral studies

To produce acute and persistent inflammatory pain, carrageenan (2% lambda carrageenan in saline, 25 μl, Sigma, St. Louis, MO) and complete Freund’s adjuvant (CFA, 25 μl, Sigma) were injected into the plantar surface of the right hindpaw under light halothane anesthesia, respectively [16,51-54]. Control mice were treated with saline or incomplete Freund’s adjuvant (IFA, Sigma), respectively.

To produce peripheral neuropathic pain, spinal nerve ligation (SNL) was carried out as described [44,55]. For sham surgery, mice were treated similarly except the spinal process was not removed to avoid damage to the underlying spinal nerves.

Mechanical allodynia and thermal hyperalgesia were measured using the Dynamic Plantar Aesthesiometer (Ugo Basile, Comerio VA, Italy) and the Paw Thermal Stimulator (UCSD, San Diego, CA, USA), respectively as described [16,44]. In CFA model, these behavioral experiments were conducted 3 days after the injection, and in SNL model, the behavioral experiments were conducted between 2 to 3 weeks after the operation. Intrathecal (i.t.) injection was given in a volume of 5 μl by percutaneous puncture through an intervertebral space at the level of the 5th or 6th lumbar vertebra, according to a previously reported procedure [16,44,56].

For rotarod experiments, mice were placed on a rotating rod (MK-630B, Muromachi Kikai Co., Tokyo, Japan) and the latency to fall was measured. Cut off time was 300 s. The speed of rotation was accelerated from 4.0 to 40 rpm in 5 min. Each mouse underwent two baseline trials and data were averaged. Then, one test trial was performed at 1.5 h after i.t. administration of GPR40 agonists.

An investigator, who was unaware of the drug treatment, performed all of the behavioral experiments.

### Blood glucose measurement

Blood glucose was measured from tail bleeds 1.5 h after i.t. administration of GPR40 agonists or vehicle control (0.1% DMSO) using a Glutest EII (Sanwa Kagaku Kenkyusho Co., Aichi, Japan). Mice were not fasted.

### Histology

The animals were deeply anesthetized with sodium pentobarbital (60 mg/kg, i.p.) and perfused intracardially with heparinized saline followed by 4% paraformaldehyde in 0.1 M phosphate buffer (pH 7.4). After laminectomy the spinal cord and DRGs (L3-L5) were identified, excised and postfixed over night at 4°C in the same fixative, and then replaced with 30% sucrose in 0.1 M phosphate buffered saline (PBS) at 4°C for cryoprotection. Transverse spinal and DRG sections (8 μm) were cut on a cryostat and collected on MAS-coated glass slide (Matsunami glass, Japan). Sections of a set of control and experimental tissues were concurrently immunostained and images were captured under the similar conditions.

### Single immunostaining

Cryosections were incubated in methanol containing 0.3% hydrogen peroxide to block endogenous peroxidase activity, then in 10 mM citrate buffer (pH 6.0) at 85°C for 20 min. Sections were blocked in PBS containing 5% normal horse serum for 60 min at room temperature, and incubated over night at 4°C with the primary antibodies against GPR40 (goat, 1:50, Y-17, sc-28416, Santa Cruz or goat, 1:150, C-17, sc-28417, Santa Cruz). Antibody detection was performed by incubating slices for 120 min with biotinylated horse anti-goat IgG (1:200, VA-9500, Vector, Burlingame, CA) as a secondary antibody, followed by VECTASTAIN ABC (Vector) for 60 min, and then with 3,3′-diaminobenzidine complex (Vector). Single immunostaining was detected using a microscope (AX70, Olympus, Japan). The specificity of antibodies was checked by pre-absorption with antigen peptide (sc-28416P for Y-17 and sc-28417P for C-17, Santa Cruz).

### Double immunostaining

Cryosections were incubated in 10 mM citrate buffer (pH 6.0) at 85°C for 20 min, then blocked in PBS containing 5% normal horse serum for 60 min at room temperature. Sections were incubated over night at 4°C with the primary antibodies against GPR40 (goat, 1:50, Y-17, sc-28416, Santa Cruz or goat, 1:150, C-17, sc-28417, Santa Cruz). After washed with PBS three times, the sections were incubated for 2 h at room temperature with Alexa Fluor 546-labeled donkey anti-goat IgG (1:400, A11056, Life Technologies, Carlsbad, CA). Sections were washed three times in PBS, followed by incubation with antibodies against cell type-specific markers; neuronal specific nuclear protein (NeuN; mouse, 1:1000; MAB377; Millipore), glial fibrillary acid protein (GFAP; rabbit, 1:10, N1506, Dako, Carpentaria, CA) and ionized calcium binding adaptor molecule 1 (Iba 1, rabbit, 1:1000, 019–19741, Wako, Japan). After washed with PBS three times, the sections were incubated for 2 h at room temperature with Alexa Fluor 488-labeled goat anti-mouse IgG (1:400, A11054, Life Technologies) or Alexa Fluor 488-labeled goat anti-rabbit IgG (1:400, A11029, Life Technologies). At the end of the staining periods, the sections were counterstained with 4′,6-diamidino-2-phenylindole (DAPI) and observed with the aid of a fluorescence microscope (AXIO Imager Z1, Carl Zeiss, Germany) for Figure 2 or a confocal microscope (A1, Nikon) for Figures 4 and 5.

### Immunoblot analysis

Six hours after carrageenan or saline injection, 3 days after CFA or IFA injection, and 2–3 weeks after SNL or sham operation, mice were anesthetized with sodium pentobarbital (50 mg/kg, i.p.), and the lumbar spinal cord and dorsal root ganglia (DRGs) (L3-L5) were quickly removed. For naïve control, whole spinal cord was used. In the pain models and their controls, ipsilateral or both ipsilateral and contralateral sides to the vehicle injection, inflammation, or operation were used. Each sample was homogenized in a lysis buffer [150 mM NaCl, 1 mM EDTA, 1% NP-40, 0.5% sodium deoxycholate, 0.1% SDS, and 50 mM Tris–HCl, pH 8.0] with a protease inhibitor cocktail (Roche Diagnostics, Mannheim, Germany). Protein concentrations were determined with a Bio-Rad protein assay kit (Bio-Rad, Hercules, CA). Proteins (50 μg) were separated by SDS-PAGE (12.5% gel) and then transferred to a polyvinylidene difluoride membrane (Millipore, Billerica, MA). Two kinds of anti-GPR40 antibodies were used. One was raised against a peptide mapping within an internal cytoplasmic domain of human GPR40 (goat polyclonal; 1:500; Y-17, sc-28416, Santa Cruz Biotechnology, Santa Cruz, CA), and the other was raised against a peptide mapping within a C-terminal cytoplasmic domain of human GPR40 (goat polyclonal; 1:500; C-17, sc-28417, Santa Cruz). The specificities of these two antibodies were tested with each blocking peptide (sc-28416P and sc-28417P, respectively) for competition studies.

Immunoreactivity was detected by using an ECL prime kit (GE Healthcare, Buckinghamshire, UK). An anti-β-actin antibody (mouse monoclonal, 1:1000; no. sc-47778, Santa Cruz) was used to normalize protein loading. Relative intensities of the bands were quantified by using an image analysis system with Image J software, version 1.48v (National Institutes of Health, Bethesda, MD). At least two independent immunoblot experiments of three independent spinal cord and DRG samples were analyzed.

### Patch-clamp recordings from spinal dorsal horn neurons

Adult mouse spinal cord slices were prepared according to the method of Yoshimura & Jessell [16-18,44]. Briefly, 6 hours after carrageenan or saline injection, 3 days after CFA or IFA injection, and 2–3 weeks after SNL or sham operation, transverse slices (thickness, 700–750 μm) of the L4 or 5 spinal segment with each dorsal root attached were cut on a vibrating blade slicer. We used L4 segment with the L4 dorsal root attached from L4/5 SNL and sham-operated mice to detect possible plastic changes of neighboring L3 DRG, because L4 segment would be more easily affected by L3 primary afferent input than L5 segment through rostrocaudal distribution of excitatory and inhibitory synaptic responses induced by Aδ and C primary afferent inputs [57]. The slices were superfused with Krebs solution (10–15 ml/min) saturated with 95% O_2_ and 5% CO_2_ at 36 ± 1°C. The composition of Krebs solution was as follows (in mM): NaCl 117; KCl 3.6; NaHCO_3_ 25; NaH_2_PO_4_ 1.2; CaCl_2_ 2.5; MgCl_2_ 1.2, and glucose 11 (pH 7.4 after gas saturation).

Blind whole-cell patch-clamp recordings were made from the lamina II (substantia gelatinosa: SG) neurons ipsilateral to carrageenan, CFA, SNL or control (saline, IFA or sham) treatment in voltage clamp mode. Patch pipettes were fabricated from thin-walled, borosilicate, glass-capillary tubing (1.5 mm o.d., World Precision Instruments). After establishing the whole-cell configuration, neurons were held at the potential of −70 mV to record spontaneous excitatory postsynaptic currents (sEPSCs). Under this condition, GABA- and glycine-mediated IPSCs were negligible, because the holding potential was close to the reversal potentials of IPSCs [58]. Recording electrodes were filled with potassium gluconate-based solution (in mM: K-gluconate 135; KCl 5; CaCl_2_ 0.5; MgCl_2_ 2; EGTA 5; HEPES 5; ATP-Mg 5; adjusted with KOH to pH 7.2). The resistance of a typical patch pipette is 5–10 MΩ. Membrane currents were amplified with an Axopatch 200B amplifier (Molecular Devices, Sunnyvale, CA, USA) in voltage-clamp mode. Signals were low-pass filtered at 5 kHz and digitized at 333 kHz with an A/D converter (Digidata 1322, Molecular Devices). Data were stored with a personal computer using pCLAMP10 software and analyzed with Mini Analysis software (Synaptosoft Inc., Decatur, GA, USA).

The average values of both frequency and amplitude of sEPSCs during the control (1 min period immediately before drug application) and 1 min period after the attainment of steady effect of each drug were calculated and quantified as relative changes in frequency and amplitude. Since the passive membrane properties (resting membrane potential and input membrane resistance) and the characteristics of sEPSCs parameters (frequency and amplitude) were not significantly different among naïve-, saline-, IFA- and sham-control, data from each control were combined.

### Drugs

MEDICA16, GW9508 and GW1100 were purchased from Cayman chemical company (Ann Arbor, MI, USA). These drugs were made up as concentrated stock solution in DMSO, which was purged with N_2_ gas, aliquoted and stored at −70°C. An aliquot was diluted to the desired concentration in saline or Krebs solution immediately prior to use. The maximum concentration of vehicle used to dilute drugs (0.1% DMSO) had no effect on the mechanical and thermal thresholds, and the mean frequency and amplitude of sEPSCs. The concentration of these drugs applied in the patch-clamp experiments were defined according to the effective concentration used previously by other researchers [3,12-14] and our preliminary studies.

### Statistical analysis

Experimental data are expressed as mean ± SEM. Single comparisons were made using Student’s two-tailed paired or unpaired *t*-test. One-way ANOVA followed by the Dunnett’s or Tukey’s test was used for multiple comparisons. *P* < 0.05 was considered statistically significant.

## Additional files

Additional file 1:
**Effects of GPR40 agonists on naïve mice.** Intrathecal (i.t.) injection of MEDICA16 (100 pmol; **A, B**) or GW9508 (30 pmol; **C, D**) had no effects on mechanical **(A, C)** and thermal **(B, D)** sensitivities of hindpaws. Paw withdrawal threshold to mechanical stimulation or paw withdrawal latency to thermal stimuli are plotted against the time after i.t. injection. Data from right and left hindpaws were combined and averaged in both tests. Each point and vertical bar represent the mean and SEM. Vertical bars are indicated only when larger than symbols. Additional file 2:
**Antagonistic action of GW1100 against GW9508-induced anti-hyperalgesic effect.** **(A)** Comparison between MEDICA16- and GW9508-induced anti-hyperalgesic effects. The GPR40 agonist, MEDICA16 or GW9508, was injected intrathecally (i.t.) 6 hours after carrageenan (Car) injection. **(B)** The anti-hyperalgesic effect of GW9508 was blocked by GW1100. Paw withdrawal latency to thermal stimuli are plotted against the time after carrageenan injection into a hindpaw. Data are mean ± SEM. ^*^ *P* < 0.05, ^**^ *P* < 0.01, ^***^ *P* < 0.001, compared with pre-drug (at 6 hours) data (one-way ANOVA followed by Dunnett’s post hoc test). ^#^ *P* < 0.05, ^##^ *P* < 0.01, ^###^ *P* < 0.001, compared with MEDICA16 (30 pmol) group in **(A)** and GW9508 (30 pmol) group in **(B)** (Student's *t*-test). Additional file 3:
**GPR40 agonists had no effects on motor coordination and blood glucose levels.** **(A)** Falling latency (time on rotarod) in the rotarod test and effects of MEDICA16 (100 pmol) and GW9508 (30 pmol). **(B)** Blood glucose levels after MEDICA16 (100 pmol) and GW9508 (30 pmol). Intrathecal administration of MEDICA16 and GW9508 did not show any significant effects on both motor function and blood glucose levels, although near maximum antinociceptive effects were observed at this time point (1.5 h after injection) in all three pain models (see Figure 6 and Additional file 2). Additional file 4:
**Comparison of passive membrane properties among SG neurons obtained from control, inflamed and nerve injured mice.** RMP, resting membrane potential; IMR, input membrane resistance, CFA, complete Freund’s adjuvant, SNL, spinal nerve ligation. Additional file 5:
**Effects of peripheral inflammation or spinal nerve injury on spontaneous EPSCs (sEPSCs).** Hindpaw injection of CFA but neither carrageenan (Car) nor spinal nerve ligation (SNL) injury significantly increased mean frequency of sEPSCs. CFA, Car or SNL treatment did not changed mean amplitudes of sEPSCs. Three days (CFA 3d) or 6 hours (Car 6h) after injection, or 2-3 weeks after SNL injury, spinal cord slices were prepared and blind whole-cell patch-clamp recordings were made from the SG neurons ipsilateral to Car, CFA, SNL, or their respective control treatment. ^*^ *P* < 0.05, ^**^ *P* < 0.01; one-way ANOVA followed by Tukey’s post hoc test. Additional file 6:
**GW9508 decreased the mean frequency of sEPSCs in inflammatory and neuropathic pain model mice.** Representative traces of sEPSCs in SG neurons of the spinal cord slices from control **(A)**, carrageenan **(B)**-, CFA **(C)**- and SNL **(D)**-treated mice showing the effects of GW9508 (30 μM). Lower five traces represent sEPSCs at five given points in time presented above the upper trace, and are shown in an expanded time scale. Additional file 7:
**Effects of GPR40 agonists on sEPSC amplitude.** CFA, complete Freund’s adjuvant, SNL, spinal nerve ligation. Increase or decrease means the increase or decrease of 10% or more in the sEPSC amplitude, respectively. Values are expressed as mean ± SEM (%) and the proportion of neurons exhibiting increase, decrease or no change in parentheses.^*^ *P* < 0.05, when compared with pretreatment control (Student’s *t* test). Additional file 8:
**MEDICA16 elicited outward currents at -70 mV in some SG neurons in SNL model mice.** MEDICA16 (10 μM) was applied during time indicated by the bar.

## Abbreviations

CFA: Complete Freund’s adjuvant
CNS: Central nervous system
DRG: Dorsal root ganglion
FFA: Free fatty acid
GFAP: Glial fibrillary acid protein
GPR: G-protein-coupled receptor
Iba 1: Ionized calcium binding adaptor molecule 1
IFA: Incomplete Freund’s adjuvant
IR: Immunoreactivity
i.t.: Intrathecal
Neu N: Neuronal specific nuclear protein
sEPSC: Spontaneous excitatory postsynaptic currents
SG: Substantia gelatinosa
SNL: Spinal nerve ligation
