# Supplementary material for: Attenuation of inflammatory and neuropathic pain behaviors in mice through activation of free fatty acid receptor GPR40
Source: Mol Pain. 2015 Feb 12;11:6. doi: 10.1186/s12990-015-0003-8 (PMC4339434; doi:10.1186/s12990-015-0003-8)
Supplement: Additional file 1: — Effects of GPR40 agonists on naïve mice. Intrathecal (i.t.) injection of MEDICA16 (100 pmol; A, B) or GW9508 (30 pmol; C, D) had no effects on mechanical (A, C) and thermal (B, D) sensitivities of hindpaws. Paw withdrawal threshold to mechanical stimulation or paw withdrawal latency to thermal stimuli are plotted against the time after i.t. injection. Data from right and left hindpaws were combined and averaged in both tests. Each point and vertical bar represent the mean and SEM. Vertical bars are indicated only when larger than symbols. [file 12990_2015_3_MOESM1_ESM.doc]

**Additional file 1: Effects of GPR40 agonists on naïve mice.** Intrathecal (i.t.) injection of MEDICA16 (100 pmol; **A, B**) or GW9508 (30 pmol; **C, D**) had no effects on mechanical (**A, C**) and thermal (**B, D**) sensitivities of hindpaws. Paw withdrawal threshold to mechanical stimulation or paw withdrawal latency to thermal stimuli are plotted against the time after i.t. injection. Data from right and left hindpaws were combined and averaged in both tests. Each point and vertical bar represent the mean and SEM. Vertical bars are indicated only when larger than symbols.

**Karki et al. Additional file 1**
