# Supplementary material for: Attenuation of inflammatory and neuropathic pain behaviors in mice through activation of free fatty acid receptor GPR40
Source: Mol Pain. 2015 Feb 12;11:6. doi: 10.1186/s12990-015-0003-8 (PMC4339434; doi:10.1186/s12990-015-0003-8)
Supplement: Additional file 2: — Antagonistic action of GW1100 against GW9508-induced anti-hyperalgesic effect. (A) Comparison between MEDICA16- and GW9508-induced anti-hyperalgesic effects. The GPR40 agonist, MEDICA16 or GW9508, was injected intrathecally (i.t.) 6 hours after carrageenan (Car) injection. (B) The anti-hyperalgesic effect of GW9508 was blocked by GW1100. Paw withdrawal latency to thermal stimuli are plotted against the time after carrageenan injection into a hindpaw. Data are mean ± SEM. * P < 0.05, ** P < 0.01, *** P < 0.001, compared with pre-drug (at 6 hours) data (one-way ANOVA followed by Dunnett’s post hoc test). # P < 0.05, ## P < 0.01, ### P < 0.001, compared with MEDICA16 (30 pmol) group in (A) and GW9508 (30 pmol) group in (B) (Student's t-test). [file 12990_2015_3_MOESM2_ESM.doc]

**Additional file 2: Antagonistic action of GW1100 against GW9508-induced anti-hyperalgesic effect.** (A) Comparison between MEDICA16- and GW9508-induced anti-hyperalgesic effects. The GPR40 agonist, MEDICA16 or GW9508, was injected intrathecally (i.t.) 6 hours after carrageenan (Car) injection. (B) The anti-hyperalgesic effect of GW9508 was blocked by GW1100. Paw withdrawal latency to thermal stimuli are plotted against the time after carrageenan injection into a hindpaw. Data are mean ± SEM. ^*^*P* < 0.05, ^**^*P* < 0.01, ^***^*P* < 0.001, compared with pre-drug (at 6 hours) data (one-way ANOVA followed by Dunnett’s post hoc test). ^#^*P* < 0.05, ^##^*P* < 0.01, ^###^*P* < 0.001, compared with MEDICA16 (30 pmol) group in (A) and GW9508 (30 pmol) group in (B) (Student's t-test).
