# Supplementary material for: Attenuation of inflammatory and neuropathic pain behaviors in mice through activation of free fatty acid receptor GPR40
Source: Mol Pain. 2015 Feb 12;11:6. doi: 10.1186/s12990-015-0003-8 (PMC4339434; doi:10.1186/s12990-015-0003-8)
Supplement: Additional file 3: — GPR40 agonists had no effects on motor coordination and blood glucose levels. (A) Falling latency (time on rotarod) in the rotarod test and effects of MEDICA16 (100 pmol) and GW9508 (30 pmol). (B) Blood glucose levels after MEDICA16 (100 pmol) and GW9508 (30 pmol). Intrathecal administration of MEDICA16 and GW9508 did not show any significant effects on both motor function and blood glucose levels, although near maximum antinociceptive effects were observed at this time point (1.5 h after injection) in all three pain models (see Figure 6 and Additional file 2). [file 12990_2015_3_MOESM3_ESM.doc]

**Additional file 3: GPR40 agonists had no effects on motor coordination and blood glucose levels.** (**A**) Falling latency (time on rotarod) in the rotarod test and effects of MEDICA16 (100 pmol) and GW9508 (30 pmol). (**B**) Blood glucose levels after MEDICA16 (100 pmol) and GW9508 (30 pmol). Intrathecal administration of MEDICA16 and GW9508 did not show any significant effects on both motor function and blood glucose levels, although near maximum antinociceptive effects were observed at this time point (1.5 h after injection) in all three pain models (see Figure 6 and Additional file 2).

**Karki et al. Additional file 3**
