# Supplementary material for: Attenuation of inflammatory and neuropathic pain behaviors in mice through activation of free fatty acid receptor GPR40
Source: Mol Pain. 2015 Feb 12;11:6. doi: 10.1186/s12990-015-0003-8 (PMC4339434; doi:10.1186/s12990-015-0003-8)
Supplement: Additional file 4: — Comparison of passive membrane properties among SG neurons obtained from control, inflamed and nerve injured mice. RMP, resting membrane potential; IMR, input membrane resistance, CFA, complete Freund’s adjuvant, SNL, spinal nerve ligation. [file 12990_2015_3_MOESM4_ESM.doc]

**Additional file 4: Comparison of passive membrane properties among SG neurons obtained from control, inflamed and nerve injured mice.**

|  | RMP (mV) | IMR (MΩ) |
| --- | --- | --- |
| Control | - 63.7 ± 1.6 (n = 18) | 614.5 ± 49.4 (n = 16) |
| Carrageenan 6 h | - 64.8 ± 2.0 (n = 14) | 679.8 ± 53.4 (n = 13) |
| CFA 3d | - 62.1 ± 1.0 (n = 36) | 573.1 ± 50.2 (n = 28) |
| SNL 2 ~ 3 w | - 65.0 ± 1.8 (n = 33) | 652.8 ± 39.7 (n = 31) |

RMP, resting membrane potential; IMR, input membrane resistance, CFA, complete Freund’s adjuvant, SNL, spinal nerve ligation.

**Karki et al. Additional file 4**
