# Supplementary material for: Attenuation of inflammatory and neuropathic pain behaviors in mice through activation of free fatty acid receptor GPR40
Source: Mol Pain. 2015 Feb 12;11:6. doi: 10.1186/s12990-015-0003-8 (PMC4339434; doi:10.1186/s12990-015-0003-8)
Supplement: Additional file 5: — Effects of peripheral inflammation or spinal nerve injury on spontaneous EPSCs (sEPSCs). Hindpaw injection of CFA but neither carrageenan (Car) nor spinal nerve ligation (SNL) injury significantly increased mean frequency of sEPSCs. CFA, Car or SNL treatment did not changed mean amplitudes of sEPSCs. Three days (CFA 3d) or 6 hours (Car 6h) after injection, or 2-3 weeks after SNL injury, spinal cord slices were prepared and blind whole-cell patch-clamp recordings were made from the SG neurons ipsilateral to Car, CFA, SNL, or their respective control treatment. * P < 0.05, ** P < 0.01; one-way ANOVA followed by Tukey’s post hoc test. [file 12990_2015_3_MOESM5_ESM.doc]

**Additional file 5: Effects of peripheral inflammation or spinal nerve injury on spontaneous EPSCs (sEPSCs).** Hindpaw injection of CFA but neither carrageenan (Car) nor spinal nerve ligation (SNL) injury significantly increased mean frequency of sEPSCs. CFA, Car or SNL treatment did not changed mean amplitudes of sEPSCs. Three days (CFA 3d) or 6 hours (Car 6h) after injection, or 2-3 weeks after SNL injury, spinal cord slices were prepared and blind whole-cell patch-clamp recordings were made from the SG neurons ipsilateral to Car, CFA, SNL, or their respective control treatment. ^*^*P* < 0.05, ^**^*P* < 0.01; one-way ANOVA followed by Tukey’s post hoc test.
