# Supplementary material for: Attenuation of inflammatory and neuropathic pain behaviors in mice through activation of free fatty acid receptor GPR40
Source: Mol Pain. 2015 Feb 12;11:6. doi: 10.1186/s12990-015-0003-8 (PMC4339434; doi:10.1186/s12990-015-0003-8)
Supplement: Additional file 6: — GW9508 decreased the mean frequency of sEPSCs in inflammatory and neuropathic pain model mice. Representative traces of sEPSCs in SG neurons of the spinal cord slices from control (A), carrageenan (B)-, CFA (C)- and SNL (D)-treated mice showing the effects of GW9508 (30 μM). Lower five traces represent sEPSCs at five given points in time presented above the upper trace, and are shown in an expanded time scale. [file 12990_2015_3_MOESM6_ESM.doc]

**Additional file 6: GW9508 decreased the mean frequency of sEPSCs in inflammatory and neuropathic pain model mice.** Representative traces of sEPSCs in SG neurons of the spinal cord slices from control **(A)**, carrageenan **(B)**-, CFA **(C)**- and SNL **(D)**-treated mice showing the effects of GW9508 (30 μM). Lower five traces represent sEPSCs at five given points in time presented above the upper trace, and are shown in an expanded time scale.

**Karki et al. Additional file 6**
