# Supplementary material for: Attenuation of inflammatory and neuropathic pain behaviors in mice through activation of free fatty acid receptor GPR40
Source: Mol Pain. 2015 Feb 12;11:6. doi: 10.1186/s12990-015-0003-8 (PMC4339434; doi:10.1186/s12990-015-0003-8)
Supplement: Additional file 7: — Effects of GPR40 agonists on sEPSC amplitude. CFA, complete Freund’s adjuvant, SNL, spinal nerve ligation. Increase or decrease means the increase or decrease of 10% or more in the sEPSC amplitude, respectively. Values are expressed as mean ± SEM (%) and the proportion of neurons exhibiting increase, decrease or no change in parentheses.* P < 0.05, when compared with pretreatment control (Student’s t test). [file 12990_2015_3_MOESM7_ESM.doc]

**Additional file 7: Effects of GPR40 agonists on sEPSC amplitude.**

|  | **MEDICA16 (10 μM)** | | |  | **GW9508 (30 μM)** | | |  |
| --- | --- | --- | --- | --- | --- | --- | --- | --- |
|  | Increase | Decrease | No change |  | Increase | Decrease | No change |  |
| **Control** | 130.0 (1/21) | 84.2 ± 2.0^*^(3/21) | 100.4 ± 1.2 (17/21) |  | 119.4 ± 1.4 (2/8) | 89.8 (1/8) | 97.3 ± 2.1 (5/8) |  |
| **Carrageenan 6h** | ― (0/8) | 88.6 (1/8) | 102.9 ± 2.4 (7/8) |  | 118 (1/8) | 75.0 ± 2.1 (2/8) | 98.2 ± 2.4 (5/8) |  |
| **CFA 3d** | 117.1 ± 2.3^*^ (3/15) | 85.6 ± 3.4 (3/15) | 97.6 ± 2.4 (9/15) |  | ― (0/14) | 86.7 (1/14) | 99.8 ± 1.5 (13/14) |  |
| **SNL 2-3 w** | 121.4 ± 5.5^*^ (4/15) | 74.1(1/15) | 101.3 ± 1.9 (10/15) |  | 117.2 (1/7) | 86.0 (1/7) | 97.7 ± 2.0 (5/7) |  |

CFA, complete Freund’s adjuvant, SNL, spinal nerve ligation.

Increase or decrease means the increase or decrease of 10 % or more in the sEPSC amplitude, respectively.

Values are expressed as mean ± SEM (%) and the proportion of neurons exhibiting increase, decrease or no change in parentheses.

^*^*P* < 0.05, when compared with pretreatment control (Student’s t test).

**Karki et al. Additional file 7**
