# Supplementary material for: Attenuation of inflammatory and neuropathic pain behaviors in mice through activation of free fatty acid receptor GPR40
Source: Mol Pain. 2015 Feb 12;11:6. doi: 10.1186/s12990-015-0003-8 (PMC4339434; doi:10.1186/s12990-015-0003-8)
Supplement: Additional file 8: — MEDICA16 elicited outward currents at -70 mV in some SG neurons in SNL model mice. MEDICA16 (10 μM) was applied during time indicated by the bar. [file 12990_2015_3_MOESM8_ESM.doc]

**Additional file 8: MEDICA16 elicited outward currents at -70 mV in some SG neurons in SNL model mice.** MEDICA16 (10 μM) was applied during time indicated by the bar.
